# Supplementary material for: Glutathione transferase P1 is modified by palmitate
Source: PLoS One. 2024 Sep 13;19(9):e0308500. doi: 10.1371/journal.pone.0308500 (PMC11398671; doi:10.1371/journal.pone.0308500)
Supplement: S1 Raw images — (PDF) [file pone.0308500.s001.pdf]

Marensi et al., Fig 1

Panel A

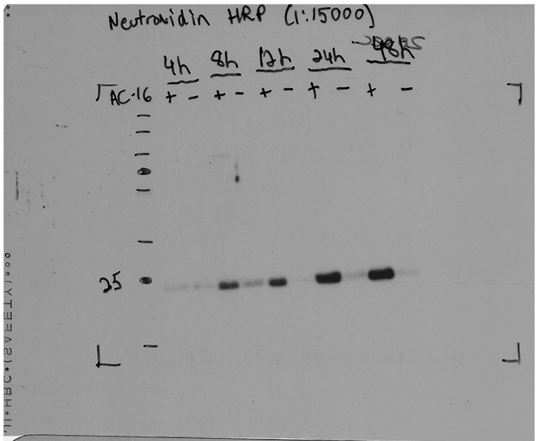

Top panel 1A

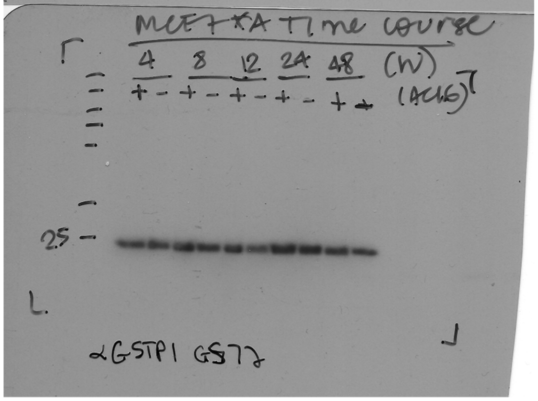

Bottom panel 1A

Panel C

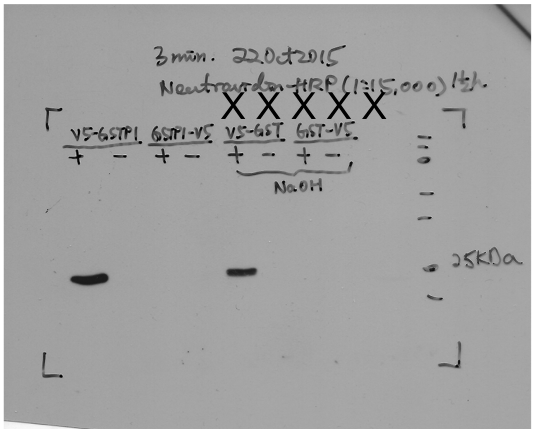

Top panel 1C

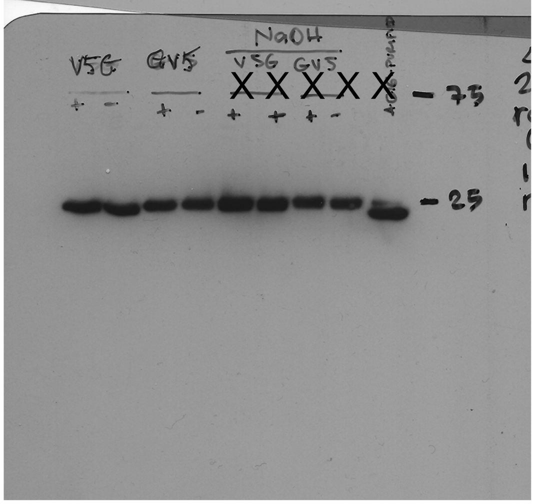

Bottom panel 1C

Marensi et al., Fig 3B

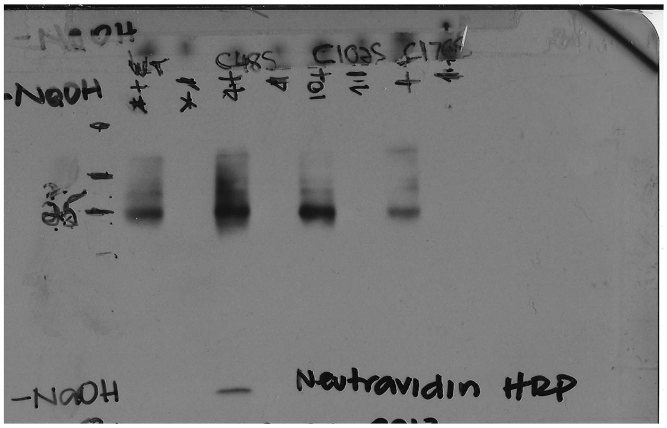

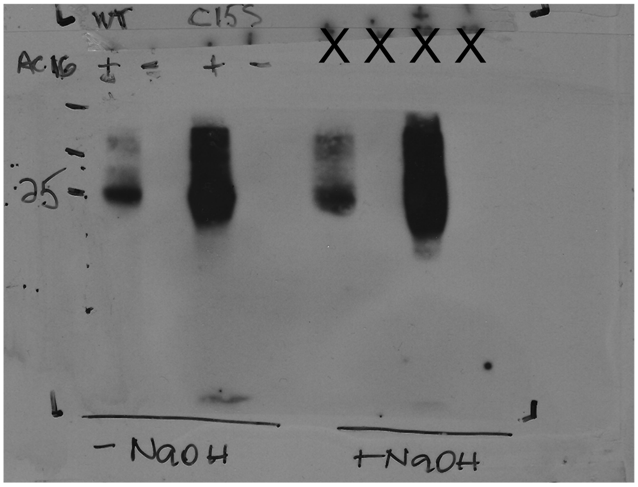

Top

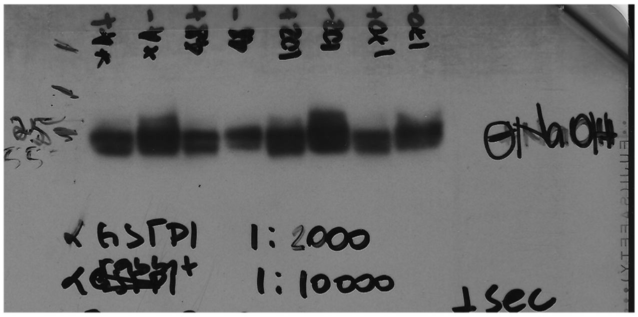

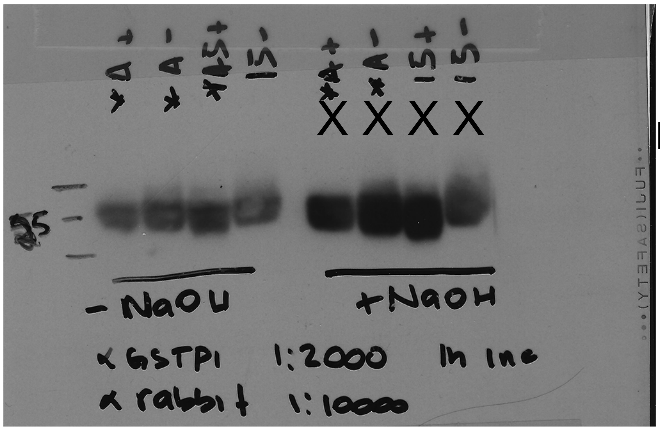

Bottom

Marensi et al., Fig 3C

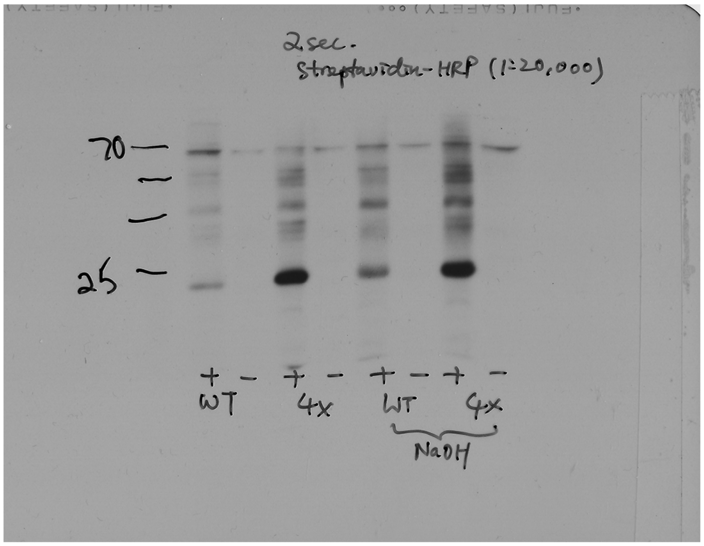

Top panel

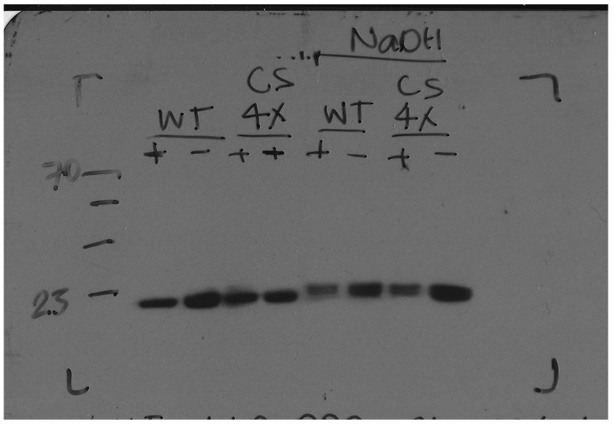

Bottom panel

Marensi et al., Fig 4A

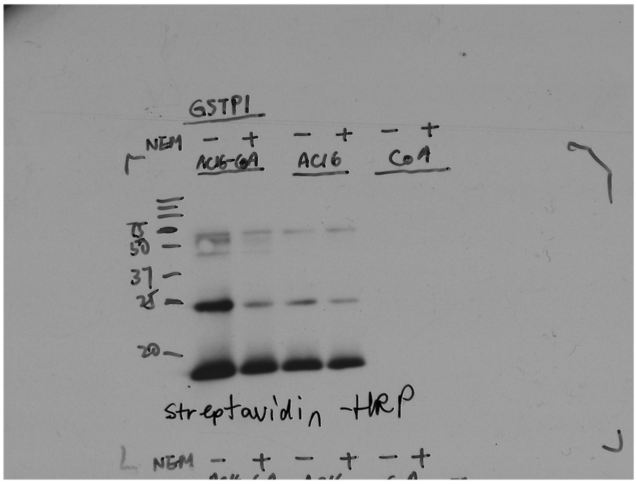

Top panel

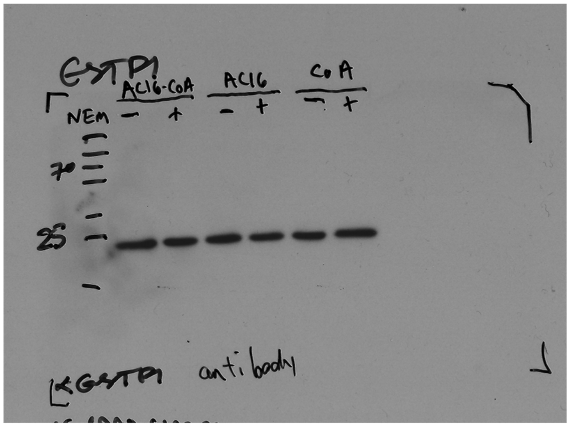

Bottom panel

Marensi et al., Fig 4B

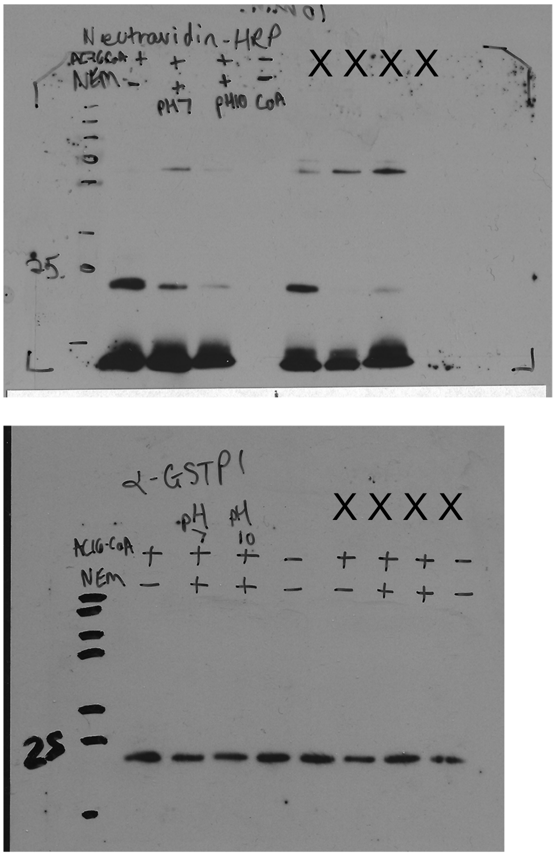

Marensi et al., Fig 4C

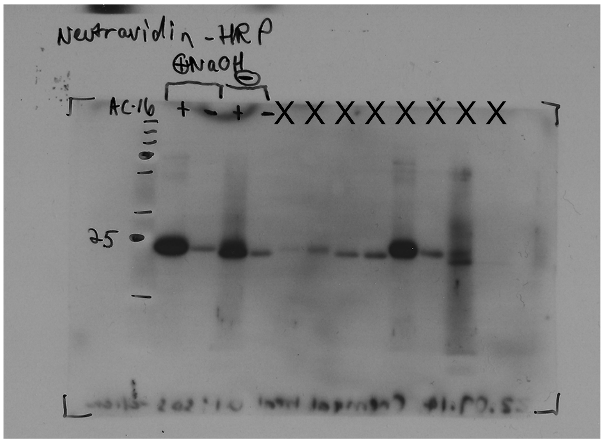

Top panel

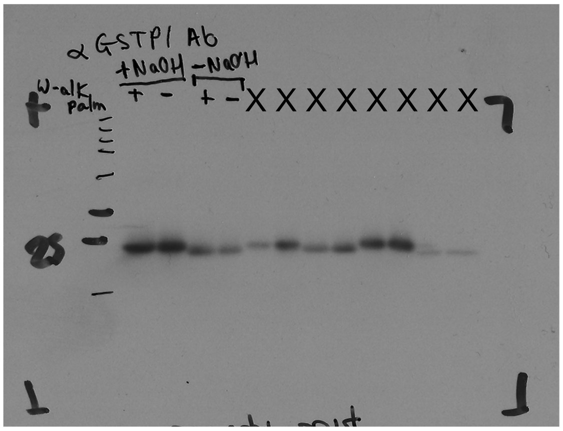

Bottom panel

Marensi et al., Fig 6A

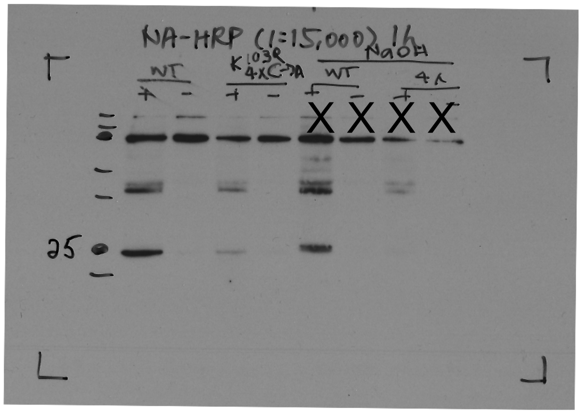

Top panel

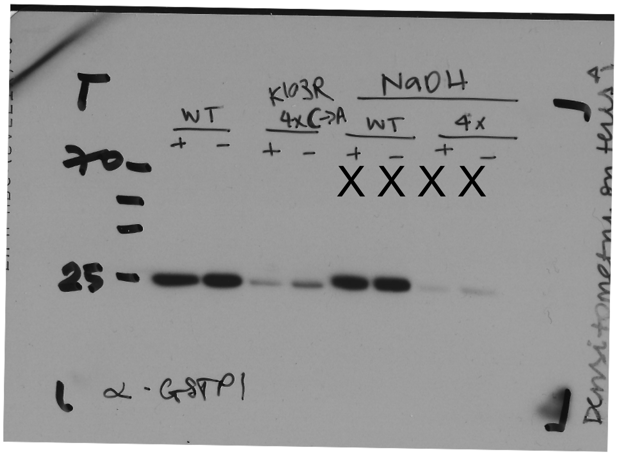

Bottom panel

Marensi et al., Fig 7A and C

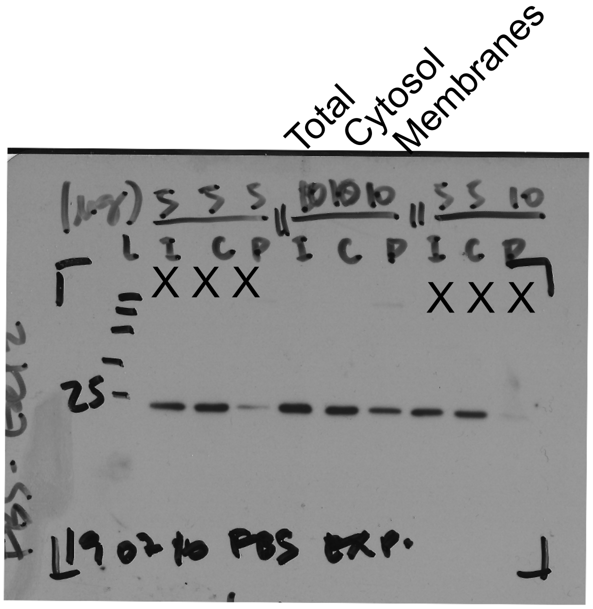

Fig 7A

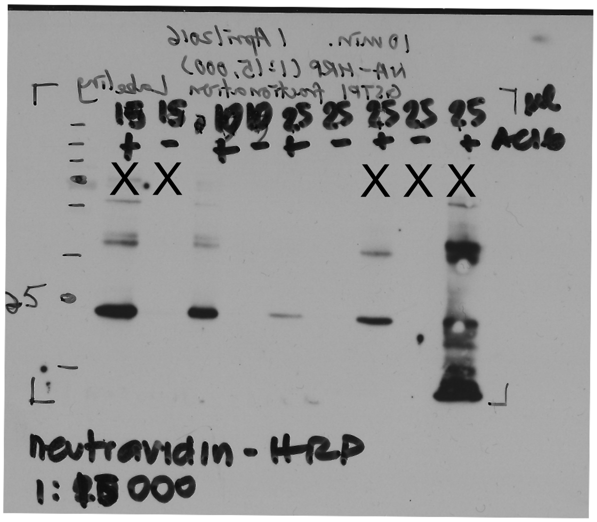

Fig 7C top panel

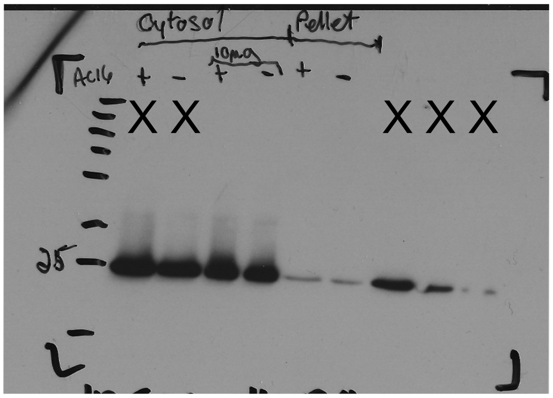

Fig 7C bottom panel

Figure 8A unstripped blot

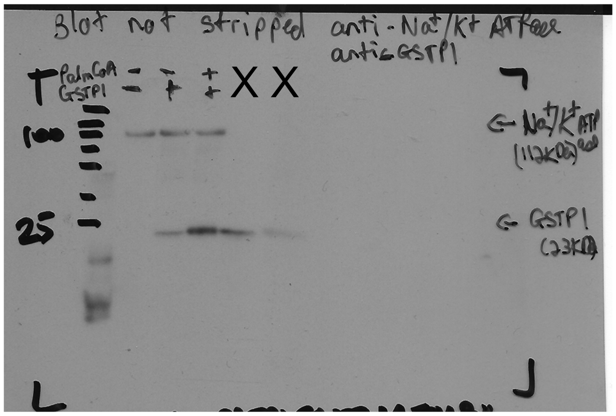

anti-Na+/K+-ATPase (bottom panel Fig 8A)

anti-GSTP1 (top panel Fig 8A)

Figure 8C unstripped blot

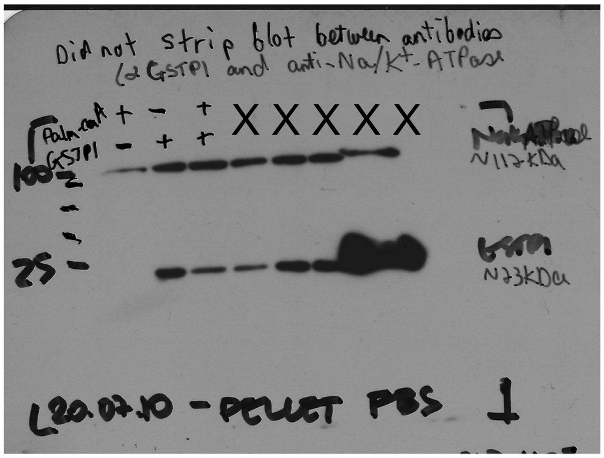

anti-Na+/K+-ATPase (bottom panel Fig 8C)

anti-GSTP1 (top panel Fig 8C)

Marensi et al.,Fig 8E

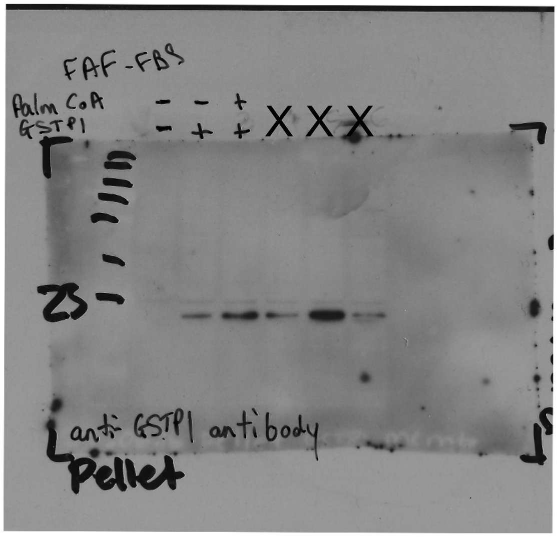

Top panel

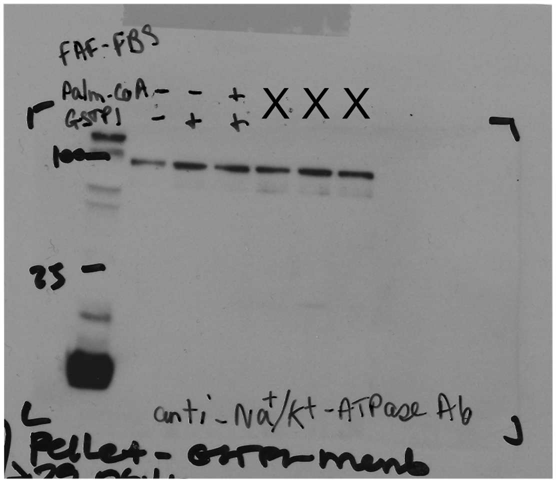

Bottom panel
