# Supplementary material for: Glutathione transferase P1 is modified by palmitate
Source: PLoS One. 2024 Sep 13;19(9):e0308500. doi: 10.1371/journal.pone.0308500 (PMC11398671; doi:10.1371/journal.pone.0308500)
Supplement: S1 File — (PDF) [file pone.0308500.s002.pdf]

Fig 1B Data set

Time (h)	% of 48 h		
**4**	0.91	0.71	0.24
**8**	18.2	23.3	16.1
**12**	78.6	33.0	45.1
**24**	68	92	100
**48**	100	100	100

Fig 6B Data set

	Neutravidin/total GSTP1	
**WT**	1	1
**4XCys to Ala/Lys103Arg**	0.78	0.8

Fig 7B data set

	Relative to cytosol				
**Total**	0.94	0.87	0.92	0.96	0.84
**Cytosol**	1	1	1	1	1
**Membranes**	0.36	0.56	0.38	0.25	0.27

Fig 7D Data set

	GSTP1 activity (nmol DNP-SG/mg/min)		
MCF7-vector			
**Total**	0.027	0.024	0.024
**Cytosol**	0.022	0.023	0.023
**Membranes**	0.025	0.021	0.021
MCF7-GSTP1			
**Total**	0.21	0.20	0.20
**Cytosol**	0.26	0.26	0.26
**Membranes**	0.022	0.020	0.022

Fig 8B Data set

	GSTP1 level relative to Na^+/K^+-ATPase			
**Vesicles alone**	0.0004	0.007	0.038	0.00015
**Vesicles + GSTP1**	0.68	0.79	0.48	0.53
**Vesicles + GSTP1 + palmitoyl CoA**	0.79	0.83	1.24	0.57

Fig 8D Data set

	**GSTP1 level relative to Na^+/K^+-ATPase**			
**Vesicles alone**	0.015	0	0.13	0.011
**Vesicles + GSTP1**	0.68	1.0	0.51	1.0
**Vesicles + GSTP1 + palmitoyl CoA**	1	0.55	1	0.17

Fig 8F Data set

	**GSTP1 level relative to Na^+/K^+-ATPase**			
**Vesicles alone**	0.003	0.007	0.022	0
**Vesicles + GSTP1**	1.0	0.72	0.41	0.53
**Vesicles + GSTP1 + palmitoyl CoA**	0.65	0.85	1.0	1.1
